# A Rapid Qualitative Screening Method for Isoniazid Tablets Using Handheld NIR Spectrometers in Two Countries

**DOI:** 10.3390/molecules28124758

**Published:** 2023-06-14

**Authors:** Matthew Eady, Jonelle Caison, Mohammed Jinnah, David Jenkins

**Affiliations:** 1 FHI 360—Product Quality Compliance Department, Durham, NC 27713, USA; 2 Department of Pharmaceutical Sciences, Campbell University, Buies Creek, NC 27506, USA; 3 FHI 360—Product Quality Compliance Department, Centurion 0157, South Africa

**Keywords:** diffuse reflectance, global health, isoniazid, near-infrared, portable spectroscopy, quality compliance, rapid testing, supply chain, tuberculosis

## Abstract

Background: Isoniazid is a leading tuberculosis treating medication. Global supply chains provide essential medicines such as isoniazid to resource-limited areas. Ensuring the safety and efficaciousness of these medicines is essential to public health programs. Handheld spectrometers are becoming increasingly approachable in cost and usability. As supply chains expand, quality compliance screening of essential medications is necessary in site-specific locations. Here, a brand-specific qualitative discrimination analysis of isoniazid is approached by collecting data from two handheld spectrometers in two countries with the intent to build a multi-location quality compliance screening method for a brand of isoniazid. Methods: Two handheld spectrometers (900–1700 nm) were used to collect spectra from five manufacturing sources (N = 482) in Durham, North Carolina, USA, and Centurion, South Africa. A qualitative brand differentiation method was established from both locations by applying a Mahalanobis distance thresholding method as a measure of assessing similarity. Results: Combining data from both locations resulted in a 100% classification accuracy, at both locations, for brand ‘A’ and resulted in the four other brands classifying as dissimilar. Bias was found between sensors in terms of resulting Mahalanobis distances, but the classification method proved to be robust enough to accommodate. Several spectral peaks found in isoniazid references appear within the 900–1700 nm range, as well as variation in the excipients per manufacturer. Conclusions: Results show promise for compliance screening isoniazid as well as other tablets in multiple geographic locations using handheld spectrometers.

## 1. Introduction

Tuberculosis is transmitted through the respiratory route by the causative agent, *Mycobacterium tuberculosis*. It is estimated that one-quarter of the world’s population is infected with tuberculosis, and that 5–15% of those cases are active [1]. Annually, it is estimated that approximately 10 million people become ill with tuberculosis, resulting in approximately 1.5 million deaths [1]. Tuberculosis is a curable disease, but difficulties in accessing health care in resource-limited areas impede treatment [2]. Medications such as isoniazid are a common treatment for tuberculosis infections and have been applied in treatment plans for decades [3].

Quality compliance monitoring of isoniazid is essential to ensuring the efficacy and safety of this essential medication to those who are in need. Traditionally, tablets such as isoniazid are tested with high-performance liquid chromatography (HPLC), which can be time-consuming and require expensive reagents, becoming a hurdle in application within limited-resource areas. Diffuse reflectance spectroscopy (DRS) has a long history of pharmaceutical process analytical technology (PAT) usage [4]. Particularly in the NIR range (relative to Raman and mid-IR), DRS can be sensitive to properties such as moisture levels, particle size, and hardness. Most of the application has centered around raw ingredient compliance validation and mixing steps [5]. Little is published on DRS as a quality compliance screening step used to supplement traditional compliance assessment methods for finished pharmaceutical products.

In recent years, ultra-portable and handheld spectrometers have entered the consumer marketplace at much lower cost points than traditional benchtop spectrometers [6]. While these handheld spectrometers typically have a reduced spectral range and bandwidth, resulting in a broader optical resolution and fewer data points collected, they have previously shown promise in both qualitative and quantitative screening methods for a variety of media, with current applications in the fields of pharmaceuticals, agri-food, forensics, soil, textiles and polymers, and fuel [7,8,9,10]. Global public health distribution networks such as the Global Health Supply Chain (GHSC) program implemented by the United States Agency for International Development (USAID) works to assist limited-resource countries (LRCs) by supplying essential medications [11]. Developing a low-cost field verification method for screening essential medicines in LRCs would assist in making sure that those medications are benefiting at-risk individuals.

A challenge in the practical implementation of spectroscopy-based screening methods is establishing calibration methods that can take into consideration naturally occurring variance between spectrometers themselves, scanning environmental conditions, and spectrometer operators [12]. In moving towards a rapid and non-destructive field-based screening method that is cost-effective, practical, and robust, a qualitative screening method needs to be established from more than a single handheld spectrometer applied at one location. Previously, our work showed that a qualitative compliance screening method could be established to differentiate between brands of depot-medroxyprogesterone acetate, an essential medication in reproductive health and hormonal therapies, using handheld spectrometers [13]. The next step is to establish a broader product compliance screening method with two spectrometers collecting data in multiple locations. Here, the objective is to determine if a qualitative screening method for differentiating between five brands of oral dosage isoniazid tablets can be established using two handheld spectrometers collecting data in Durham, North Carolina, United States, and Centurion, South Africa. Efficient qualitative screening models verified with multi-locational data would be a step towards implementing rapid spectral-based screening methods for global supply chain product assessments conducted in the field or point of use. By applying multiple handheld spectrometers as a multi-location screening method, global supply chains can more readily ensure product compliance of essential medications at a lower cost point and with faster turnaround times.

## 2. Results

### 2.1. Background Selection and Spectra

**Figure 1:**
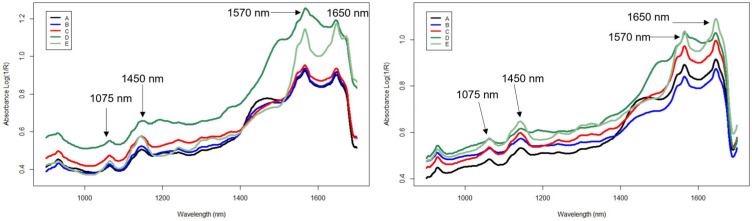
Mean spectra from five brands of isoniazid tablets collected in (**left**): Centurion, South Africa and (**right**): Durham, NC, USA using handheld spectrometers (900–1700 nm) and using background (C), a borosilicate glass vial with a Teflon insert.

Three background scanning setups were explored to determine which background setup resulted in an optimal spectrum for the tablets with the two handheld spectrometers. Background C consisted of the same borosilicate glass vials as used for sample collection, and the same 1.6 mm thick Teflon insert as the sample holder, with the only difference being that there is no center hole cut for the tablets. Background A was established to mimic the benchtop spectrometer’s setup with a custom sample holder and Spectralon cover, but the slight differences in geometric orientations of the Teflon cover made this difficult between multiple sensors. Background B was a single piece of Teflon used as a background, but was deemed inappropriate for this project, because the influence of the glass is not incorporated. The mean Absorbance Log(1/R) spectra were calculated and plotted for each of the five brands (brands A–E) of isoniazid and are shown in Figure 1, with Centurion, South Africa shown in the left panel and Durham, North Carolina, USA shown in the right panel. There is consistency between peak positions between scanning locations and across all five brands at 1570 and 1650 nm.

### 2.2. Principal Component Analysis

**Figure 2:**
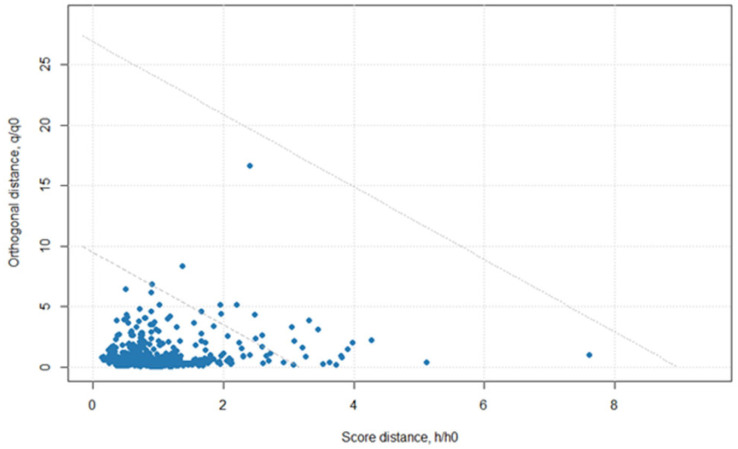
Residuals from a principal component analysis using isoniazid tablet data collected at both locations, Centurion, South Africa, and Durham, North Carolina, USA, with a handheld spectrometer (900–1700 nm).

Principal component analysis (PCA) was first applied to the entire dataset for data visualization and to observe both inter-cluster and intra-cluster sample distribution trends. The singular value decomposition (SVD)-based PCA was selected because there were more variables than observations in the reference dataset, resulting in the covariance matrix not having full rank [14]. This PCA resulted in 99.02% of the model’s explained variance within the first five PCs. Table 1 describes both the proportional and cumulative variance explained by each of the first five PCs. As common with spectroscopy datasets, the first PC explains a significant amount of the variance, i.e., 75.82%. Here, the residuals at the fifth PC were assessed as an inference check and outlier detection. Residuals were plotted as score distance (h/h_0_) by orthogonal distance (q/q_0_) for each sample and are shown in Figure 2. The dashed lines running diagonally through the plot are warning and outlier thresholds. We see that there are several samples appearing in the warning area between the dashed lines, but there are not any samples appearing above both dashed lines, which would indicate clear outliers. In order to create a robust prediction model across more than one scanning location and condition, the samples identified as warnings were left in the dataset. Thus, no samples were removed as outliers during classification.

**Figure 3:**
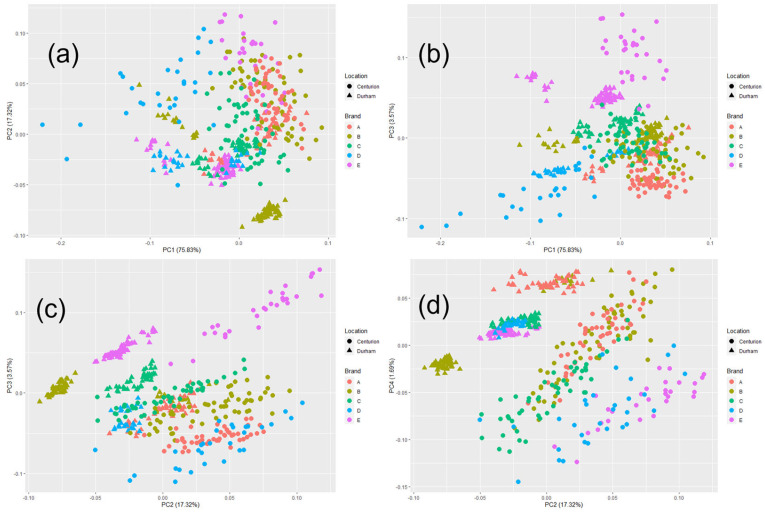
Score plots from a singular value decomposition-based principal component analysis of all samples (N = 482) collected in Centurion, South Africa and Durham, North Carolina, USA from five isoniazid tablet manufacturing sources. (**a**) Principal components (PC) 1 vs. PC 2, (**b**) PC 1 vs. PC 3, (**c**) PC 2 vs. PC 3, and (**d**) PC 2 vs. PC 4.

The PCA scores are shown for all samples and both locations (N = 482) in Figure 3. PC 1 vs. PC 2 is shown in (a), PC 1 vs. PC 3 is shown in (b), PC 2 vs. PC 3 is shown in (c), and PC 2 vs. PC 4 is shown in (d). In general, there appears to be greater intra-cluster spread with the data points collected in Centurion, South Africa, while the datapoints in Durham, North Carolina, USA appear to have less intra-cluster spread. PCA scores are always relative to what is included and is not included in the model. While one location’s variance may be greater than another, these would likely differ if other brands, manufacturing sources, or combination drugs that may contain isoniazid and rifapentine (tuberculosis medication) are introduced to the model. Scores from PC 1 and PC 2 (a) appear to have overlap between brands, except for a few separating out between other brands or scanning locations. With all brands, there appears to be a difference in score positioning between locations. More consistent clustering of brands appears when plotting PC 1 and PC 3 together as shown in (b). Separation of clusters by location is evident when plotting PC 2 vs. PC 3 shown in (c) and PC 2 vs. PC 4 shown in (d), suggesting that sensor-to-sensor variations are impacting the lower PCs more so than PC 1 and PC 2.

**Figure 4:**
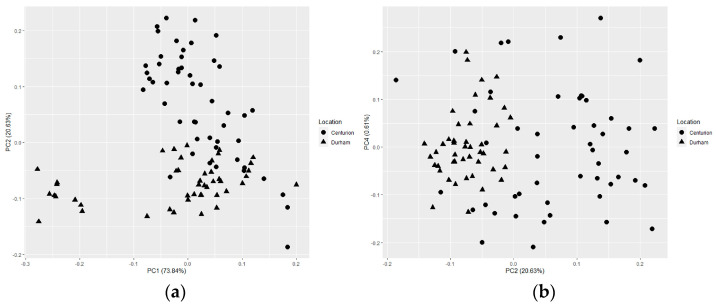
Score plots from a singular value decomposition based principal component analysis of brand ‘A’ samples used for the proceeding qualitative classification model’s reference and positive controls. Principal component (PC) 1 vs. PC 2 is shown in (**a**), while PC 2 vs. PC 4 is shown in (**b**).

Because the PCA is relative to the data input into the model, a separate SVD-based PCA was calculated using only the samples from brand ‘A’ (n = 96) to visualize similarities between data collected at both locations. These samples are used in the proceeding asymmetric multivariate classification model as the reference and positive control datasets. Here, score values from both locations are plotted to show how closely the two datasets relate. Figure 4 shows PC 1 vs. PC 2 in (a) and PC 2 vs. PC 4 in (b). While there is some overlap between samples scanned at different locations in PC 1 vs. PC 2, most of the samples are not overlapping in a normal scattering pattern. There are also samples from one lot that appear to separate themselves from the rest of the cluster on PC 1. These tablets were scanned in Durham, North Carolina, USA and not Centurion, South Africa. The variation may be attributed to a change in processes of the production run or possibly a scanning condition that varied. The lower PCs start to overlap samples scanned at the two locations a little more so, but there is still some separation between clusters of brand ‘A’ at the two locations.

**Figure 5:**
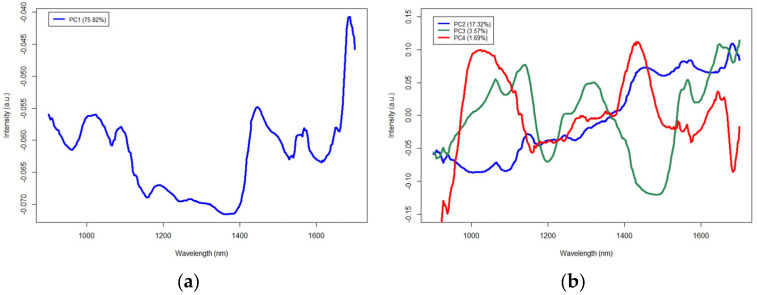
Principal component analysis loading vectors for principal components (PC) 1–4, with PC 1 shown in (**a**) and PCs 2–4 shown in (**b**). Explained variance per PC is shown as a percentage in parenthesis ().

Loading vectors were extracted from the PCA and are shown in Figure 5. PC 1 is shown in (a), while PCs 2–4 are shown in (b), due to differences in scale. NIR absorbance peaks commonly located in the range of the handheld spectrometers can be seen at 970 nm on PC 4, the peak at 1200 nm is visible on PC 1, while a third water-related NIR absorbance peak at 1450 nm can be found on PC 1 and PC 4. While moisture analyses were not conducted for this experiment, there is evidence that a difference in tablet moisture levels is present with the inter-cluster spread visible on PC 1 and PC 4. For example, brands A and E are separated on those particular PCs. Considering that there are five manufacturing sources of isoniazid, each with their own proprietary formulation methods and variations in packaging, it is likely that moisture retention by tablets could likely vary between brands. Other loading vector peaks are present and can be correlated to the active pharmaceutical ingredient, isoniazid, or potentially excipients present in the tablet formulations.

### 2.3. Spectral Peak Assignments

Loading vector peaks were extracted and correlated to spectral peaks from isoniazid, excipients, or known moisture spectra. These correlations can be seen in Table 2. Here, loading vector peaks are grouped by PC where they appeared, the corresponding reference peak, and the brand in which the spectral peak is present. This gives an indication of which variables are affecting the spectra of the five brands, and which components are contributing to that influence, as well as the PC they are contributing to. Apart from the previously mentioned moisture peaks, several significant isoniazid peaks are located along PC 1 and PC 4. There are several prominent isoniazid peaks that appear towards the end of the handheld spectrometer’s data collection range at 1643 and 1673 nm that are contributing to the PC 1 influence, while other isoniazid correlated peaks at 1065 and 1148 nm are impacting PC 4. These peaks are noticeable in the mean spectra of all five brands of isoniazid. Mannitol is an excipient only found in brand ‘D’, with correlated spectral peaks appearing on PCs 1–3. Of the five brands, brand ‘D’ appears to have the greatest PC variation and intra-cluster spread. Other common excipients such as lactose, starch, and talc have correlating peaks that appear to be prominent across PCs 1–4, and contribute in the qualitative differences between brands.

### 2.4. Two Location Classification Model

To test the ability of two handheld spectrometers to collect data of the same five brands of isoniazid tablets in two countries, a multivariate classification model for brand ‘A’ was established. By using the scores and residuals from the PCA, the dataset negates the influence of collinearity between spectra due to the orthogonal transform of the PCA. Table 3 below show the summary results of the brand ‘A’ method for each of the three backgrounds. Background C, being the borosilicate glass vial with no hole cut in the Teflon, resulted in a 100% classification accuracy from the Mahalanobis distance (M-Dist.) method. False positives were a problem for both backgrounds A and B, where many negative controls were erroneously classified as brand ‘A’. Specificity and overall accuracy improved between backgrounds A and B but were still significantly lower than needed for a rapid qualitative screening method.

A breakdown of the M-Dist. classification results can be seen in Table 4 specifically for background C, using reference files pooled into the same dataset, but results are separated based on sensors. Here, the results are listed by scanning location, but the overall method is built from all samples. Listing M-Dist. by location gives an indication as to the sensor-to-sensor variation between scanning locations. The M-Dist. classification threshold was determined to be 3.886 with five PCs selected. All four negative controls at both locations had M-Dist. values well over the threshold. NC 1 (brand ‘B’) was the closest to the reference dataset, while NC 4 (brand ‘D’) was the furthest from the reference dataset, with M-Dist. values commonly between 250 and 300. All reference and positive control samples were under the thresholds, indicating that a brand-specific model could be established between sensors collecting data in more than one location.

### 2.5. Sensor Bias

While the M-Dist. classification method showed 100% accuracy, the previous PCA score plots showed that there was some overlap between data collected at the two locations, but not a homogeneous mixture. The M-Dist. results also showed the reference dataset had mean values of 0.897 and 1.01 for Durham, North Carolina, USA and Centurion, South Africa, respectively. Because the goal here is to establish a qualitative screening method that can be applied to isoniazid tablets in various locations around the globe, a calibration transfer between handheld spectrometers would be ideal. However, these handheld spectrometers contain many optical components and hardware that can cause slight variations that may impact sensor-to-sensor comparisons, making calibration transfers difficult or not possible. Bias testing was conducted on the resulting M-Dist. scores to quantify the amount of bias between sensors, in conjunction with an asymmetrical classification model, and results are shown in Table 5. If we consider the Durham, North Carolina, USA sensor as the ‘parent’ sensor and the Centurion, South Africa sensor as the ‘child’, we calculate the SEM_Centurion_ and resulting t-value for the raw absorbance Log(1/R) data first. The raw spectra resulted in a statistically significant bias between the two sensors; therefore, preprocessing steps were applied, M-Dist. values recalculated, and the bias tests were recomputed with the new M-Dist. values. The iterative restricted least squares (IRLS) baseline-adjusted spectra also resulted in a statistically significant bias between sensors, although slightly closer t-values. Next, the multiplicative scatter correction (MSC) was applied to the dataset with results also showing a significant difference between the sensors. These results indicate that while a multi-location method for isoniazid was successfully established with data collected by two sensors in two locations, there is still bias between the sensors. The resulting asymmetrical classification model was robust enough for both sensors, although bias is still present.

## 3. Discussion

Here, two handheld spectrometers were used to collect data from isoniazid tablets obtained from five manufacturing sources. Tablets from some of the same lots were scanned in both Durham, North Carolina, USA and Centurion, South Africa. The first objective was to establish a screening method that would be appropriate for tablets. A sample holder was constructed by using a borosilicate glass vial with a Teflon insert. Affixing this to the handheld spectrometer and aligning the opening in the Teflon with the scanning window allowed for consistent data collection. Of the three background setups, the replicate vial with Teflon, but no center-cut hole, resulted in absorbance spectra that proved to be consistent between scanning locations, possibly due to slight geometric orientation differences in the top Teflon cover. Spectra between brands and locations were consistent, with notable peaks present in all mean spectra. Brands ‘D’ and ‘E’ appeared to be slightly different than the other three, but a consistent pattern was found across all.

Prior to calculating multivariate classification results, the PCA detailed visual relationships between inter- and intra-cluster variance. Because the proceeding M-Dist. classification methods was based on the orthogonal transform of the PCA scores and residuals, it was important to assess the PCA results. Some brands of isoniazid did overlap on the score plots, but not all. In general, the brands could be separated from each other visually, but also the brands by scanning location. This was also true for the PCA of only brand ‘A’ samples, which showed some overlap by location but not entirely. From the PCA scores and residuals, an M-Dist. classification method resulted in an overall accuracy of 100%, which was an improvement from the other backgrounds where specificity suffered from incorrectly identifying negative controls (brands B–E) classifying as false positives. Once the correct background was in place, no preprocessing was required for the brand ‘A’ classification method. While here we are only constructing a method for brand ‘A’, another single-class compliance screening method could just as easily be established for any of the brands ‘B’ through ‘E’ using data collected at both locations. The resulting M-Dist. values for each class were distinctly different.

Peak assignments were correlated through the collection of isoniazid and excipient spectra. These peak assignments were also correlated to the PCA loading vectors of 1 through 4. This allowed for approximating which active ingredients/excipients were influencing which PCs. Moisture peaks were noted, which is not surprising as we would expect the tablets to absorb some moisture over time during storage. Isoniazid peaks were visible and noticeable in each of the mean spectra collected at both locations, which is promising because it is the only component found in each of the five brands, as differing excipients are used.

## 4. Materials and Methods

### 4.1. Isoniazid Samples

Isoniazid tablets were collected from five manufacturing sources: Cadila Pharmaceutical Limited (Ahmendabad, India)—300 mg, Lupin Limited (Mumbai, India)—100 mg, MaCleods Pharmaceutics Limited (Mumbai, India)—300 mg, Micro Labs Limited (Karnataka, India)—300 mg, and Mylan Laboratories Limited (Hyperbad, India), with *w*/*w* % of the active pharmaceutical ingredient ranging from 49.5 to 85.5% depending on the source. In order to protect manufacturer confidentiality, results for the samples were blinded and referred to as ‘Brands A, B, C, D, and E’. Tablets from five brands were obtained from multiple production lots of each brand and scanned in either Centurion, South Africa, or at the FHI 360—Product Quality Compliance laboratory located in Durham, North Carolina, United States. Table 6 shows the breakdown of isoniazid tablets scanned by handheld spectrometers by location and brand. The total sample size was N = 482 tablets, which were scanned at two locations representing 52 unique lots obtained from five brands of isoniazid tablets. A total of 14 out of the 52 unique lots were scanned at each location, with different tablets scanned at each location for lots of the same number. A total of 220 tablets were scanned in Centurion, South Africa from 22 lots, while 262 tablets were scanned in North Carolina, USA from 44 lots.

### 4.2. Handheld Spectrometers

Tellspec Enterprise handheld spectrometers (Tellspec Inc., Toronto, ON, Canada) were used to collect data at both locations. The NIR-S-G1 spectrometers were developed by packaged by InnoSpectra (Hsinchu, Taiwan, China), and contain the DLP^®^ NIRscan Nano spectral engine (Texas Instruments, Dallas, TX, USA). Once the handheld spectrometers were received, an installation qualification and operational qualification (IQOQ) was performed at the FHI 360—PQC laboratory located in Durham, NC, USA. Initial spectrometer operating performance was evaluated, as well as conducting wavelength verification, signal-to-noise ratio, photometric linearity, and stray light assessments using NIST certified grayscale reflectivity calibration standards. Further information on the IQOQ and performance qualifications (PQs) can be found in our previous work [15].

Data collection was conducted using the freely downloaded DC&M 2.0 mobile app (Tellspec Inc., Toronto, ON, Canada), which operates through an iPhone or Android device and connects through Bluetooth to the handheld spectrometers. Scans from the sensor were initiated through the app for ease of use, as well as to avoid using the button on the scanners to minimize the movement of the sensor during tablet scanning, and to reduce the variance in the data-collection process.

### 4.3. Data Collection

**Figure 6:**
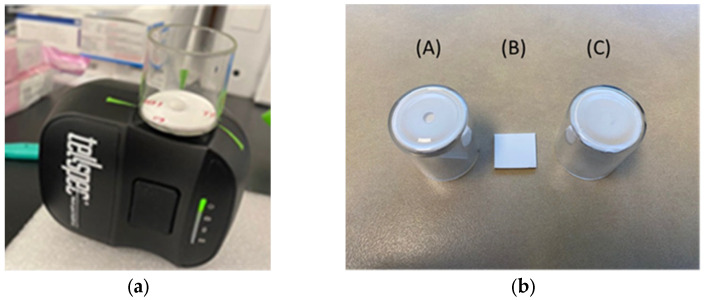
(**a**) Handheld near-infrared spectrometer setup for scanning tablets. A borosilicate glass vial with a 1.6 mm thick Teflon insert positioned directly over the spectrometer’s scanning window with a tablet placed over the opening for sample collection. (**b**) Three background setups for scanning tablets with a portable near-infrared spectrometer. (A): A borosilicate glass vial with a Teflon insert containing a 2 mm opening and a second Teflon top to cover the opening, (B): a Teflon square large enough to cover the scanning window, (C): a borosilicate glass vial with a Teflon insert and no hole.

The data-collection setup using the handheld spectrometers can be seen in Figure 6. Samples were scanned at both locations using an approximately 20 mm diameter borosilicate glass vial as a sample holder and taping this to the sensor with caution to avoid contact with the scanning window. The glass vial has a 1.6 mm thick piece of Teflon (McMaster Carr, Elmhurst, IL, USA). A 2 mm hole was cut in the center of the Teflon using a cork borer. The small opening was positioned directly on top of the spectrometer’s scanning window, to allow light to interact with the tablet placed on the 2 mm opening, while blocking ambient light. A background scan was collected using only the 1.6 mm Teflon sheeting for both locations.

Triplicate scans of the tablets were collected, rotating the tablets approximately 45° between scans. Data were exported from the spectrometers as raw reflectance values in a comma separated value (CSV) file. Raw reflectance values for the samples were adjusted by dividing the spectra of the background and converting to Absorbance Log(1/R). Triplicate scans per sample were then averaged. Additionally, isoniazid secondary standard (VWR Scientific, Radnor, PA, USA), reference spectra, and excipients (VWR Scientific, Radnor, PA, USA) were collected with a benchtop DRS (350–2500 nm) (Labspec 5000, Malvern Panalytical, Malvern, UK) in order to have a narrower band resolution of 1 nm, opposed to the variable 2–4 nm bandwidth of the handheld spectrometers. Information on which excipients were used in each brand were obtained through confidential product dossiers. Manufactured isoniazid tablet excipients by brand are shown in Table 7.

### 4.4. Data Analysis

After converting raw reflectance to Absorbance Log(1/R), data analysis was conducted in R-Studio version 4.2.1 (Boston, MA, USA). Here, spectra were analyzed, and principal component analyses (PCAs) were conducted. Packages used include ‘chemometrics’, ‘dplyr’, and ‘ggplot2’. Additionally, a PCA-based Mahalanobis distance was used as a classification method for samples, based on spectral similarities [16,17]. In brief, a reference dataset is established with samples of known good quality. PCA scores and residuals are used, with the multivariate cluster’s centroid determined. From here, new samples are projected onto the multivariate model with distance measured from each new sample to the centroid. A thresholding value is determined at a 95% confidence interval with values above the threshold classified as ‘dissimilar’ to the reference dataset and samples with M-Dist. values below the threshold classified as ‘similar’ to the reference dataset. An in-house Shiny app was developed to processes data from the handheld spectrometers, and can be found on the FHI 360—PQC GitHub site [18]. A method for classification of Product ‘A’ was established using an M-Dist. classification, where Product A was split 70/30 across both locations with 70% of the tablets used for a reference dataset and 30% used as positive controls (PCs), and the other four brands were negative controls (NCs). This is detailed in Table 8. From these results, we calculated accuracy, sensitivity, and specificity. Here, accuracy is not defined as in the Vocabulary of Metrology as measurement accuracy, but rather as the results of classifying positive and negative controls as found to be consistent with spectroscopy-based qualitative approaches found in the literature. Spectra were processed as raw Absorbance Log(1/R), as well as preprocessed with an iterative restricted least squares (IRLS) baseline correction and standard normal variant (SNV) to adjust for radiometric variance between sensors.

## 5. Conclusions

The overall classification model described here was able to incorporate enough variance from both scanning locations to result in a qualitative classification approach having 100% accuracy, while still maintaining specificity of 1.0, even though it was noted that there is a statistically significant level of bias between the two sensors. The bias between sensors is not surprising given the components that go into a handheld spectrometer and the slight instrumental variances that can occur between sensors. Preprocessing steps to normalize spectra with the MSC and baseline adjustments proved unsuccessful in reducing the bias to a level deemed statistically not significant. Therefore, future modeling with handheld spectrometers in multiple locations should likely be undertaken with data collected at both locations. Here, data were collected at both locations in near ambient conditions. Although the manufacturer specifications for these handheld spectrometers list operating conditions for the sensors at 0–40 °C and a relative humidity max of 85%, it is unclear at this time how the spectra would be affected by changes in the operating temperature or relative humidity. Future studies are planned to determine these environmental impacts. While brand-specific classification methods using isoniazid data collected in two countries resulted in 100% accuracy here, the same compliance screening principles can be applied to other essential oral medications collected in multiple locations, where there is a priority to collect data on each sensor to incorporate any sensor-to-sensor bias that may be present.

## Figures and Tables

**Table 1:** Explained variance from a principal component analysis using portable spectrometers with data collection from five brands of isoniazid tablets scanned in both Centurion, South Africa and Durham, North Carolina, USA.

Explained Variance	PC 1	PC 2	PC 3	PC 4	PC 5
Proportional	75.8	17.3	3.6	1.7	0.6
Cumulative	75.8	93.2	96.7	98.4	99.0

**Table 2:** Notable spectra peaks found in principal component analysis loading vectors and correlated to reference spectra collected on a benchtop spectrometer (350–2500 nm).

PC	Position (nm)	Reference Standard	Associated Brand Notes
1	1294 ^ψ^	Mannitol	Slightly noticeable in brand D, n/p in others
1	1429	PEG in Castor Oil	No definitive peak in brand C, n/p in others
1	1562 ^ψ^	Lactose	Definitive peak in brand C, n/p in others
1	1643 ^Υ^	Isoniazid	Sharp peak found at end of all samples
1	1673 ^Υ^	Isoniazid	Sharp peak found at end of all samples
2	979 *	Water	Moisture in tablets
2	1193	Mannitol	Slightly noticeable in brand D, n/p in others
2	1440 *	Water	Moisture in tablets
2	1635 ^ψ^	EDTA	Present in brands A and E. Not seen, but may be masked by isoniazid peak found at 1643 nm
2	1690	Mannitol/EDTA	Difficult to determine due to isoniazid peak at 1673 nm
3	1024	Unknown	Not noticeable in any mean spectra
3	1092	Unknown	Not noticeable in any mean spectra
3	1189	Soluble/Corn Starch	Possibly masked by strong isoniazid peak at 1150 nm
3	1272	Magnesium Stearate	Not noticeable in brands A and B
3	1391	Stearic Acid	Possibly overtaken by large water peak at 1440 nm
3	1510	Mannitol	Not noticeable in brand D (n/p) in others, but location is between water and isoniazid related peaks
3	1607	EDTA	Not present in brands A and E, appears to be between two strong isoniazid peaks
4	982 *	Water	Moisture in tablets
4	1065 ^Υ^	Isoniazid	Peak found in all tablets
4	1148 ^Υ^	Isoniazid	Present in all mean spectra
4	1243 ^ψ^	Lactose	Noticeable peak in brand C, n/p in others
4	1335	Unknown	Not noticeable in any mean spectra
4	1405	Talc	Possibly overtaken by large water peak at 1440 nm
4	1561 ^Υ^	Isoniazid	Noticeable in all mean spectra
4	1659 ^Υ^	Isoniazid	Noticeable in all mean spectra

* = water-associated spectral peaks, ^Υ^ = isoniazid-associated spectral peaks, and ^ψ^ = excipient-associated spectral peaks.

**Table 3:** Summary of classification results for the brand ‘A’ method using three different backgrounds, (a) a borosilicate glass vial with a Teflon insert with a 2 mm hole cut in the center and a second Teflon insert to cover the opening, (b) a single piece of Teflon, and (c) a borosilicate glass vial with a Teflon insert that had no center hole cut.

Background	Sensitivity	Specificity	Accuracy
A	1.0	0.29	40.87%
B	1.0	0.72	77.80%
C	1.0	1.0	100%

**Table 4:** Classification results for a qualitative brand ‘A’ method. Mahalanobis distance (MD) values (mean ± standard deviation) were calculated from the centroid of the reference dataset (pooled from both sensors) based on five principal component scores and residuals (99.02% explained variance).

Data	Brand	Durham MD	Durham Correct ID	S. Africa MD	S. Africa Correct ID
Ref	A	0.897 ± 0.21	32/32 (100)	1.01 ± 0.30	35/35 (100)
PC	A	0.896 ± 0.22	14/14 (100)	1.271 ± 0.49	15/15 (100)
NC 1	B	19.332 ± 4.99	69/69 (100)	9.356 ± 3.11	60/60 (100)
NC 2	C	54.608 ± 5.69	51/51 (100)	64.891 ± 7.32	50/50 (100)
NC 3	D	260.616 ± 18.37	27/27 (100)	272.61 ± 46.56	30/30 (100)
NC 4	E	26.994 ± 3.1	69/69 (100)	32.63 ± 6.45	30/30 (100)

**Table 5:** Bias testing for sensor-to-sensor comparison of two handheld spectrometers (900–1700 nm) scanning tablets in Centurion, South Africa and Durham, North Carolina, USA. Results were calculated from resulting Mahalanobis distance results per sample at each location.

Preprocessing	SEM_Centurion_	d.f.	t-Critical	t-Calculated	Bias Significant
Raw	0.0556	95	1.661	3.29	Yes
Baseline	0.0346	95	1.661	2.97	Yes
MSC	0.0462	95	1.661	2.88	Yes

MSC = multiplicative scatter correction, d.f. = degrees of freedom, SEM = standard error of the mean.

**Table 6:** Summary of lots and tablets of isoniazid scanned in Durham, North Carolina, United States and Centurion, South Africa using a handheld spectrometer (900–1700 nm).

Brand	Centurion, South Africa		Durham, NC, USA	
	# Lots	# Tablets	# Lots	# Tablets
A	5 (2)	50 (20)	8 (2)	46 (10)
B	6 (3)	60 (30)	9 (3)	69 (15)
C	5 (3)	50 (30)	9 (3)	51 (15)
D	3 (3)	30 (30)	9 (3)	27 (15)
E	3 (3)	30 (30)	9 (3)	69 (15)
Total	22 (14)	220 (140)	44 (14)	262 (70)

# = Number. ( ) = Number of lots and tablets from the same lots scanned in both locations, although these represent different tablets from the same lot numbers.

**Table 7:** List of excipients used in each brand, provided through certificates of analysis obtained through the five manufacturers blinded as A–E.

Excipient	A	B	C	D	E
calcium hydrogen phosphate	Y				
colloidal anhydrous silica/colloidal silicon dioxide	Y	Y	Y	Y	Y
croscarmellose sodium				Y	
crospovidone		Y			Y
hydrogenated castor oil			Y		
lactose monohydrate			Y		
magnesium stearate	Y	Y			Y
maize starch	Y	Y			Y
mannitol				Y	
microcrystalline cellulose		Y	Y	Y	Y
povidone				Y	
pregelatinized starch				Y	
purified talc	Y				
sodium edetate	Y				
stearic acid				Y	Y

**Table 8:** Sample breakdown of the reference, positive, and negative controls used in establishing a qualitative two-location screening method for brand A.

Brand	Type	Centurion, South Africa # Tablets	Durham, NC, USA # Tablets	Total # Tablets
A	Ref	35	32	67
A	PC	15	14	29
B	NC 1	60	69	129
C	NC 2	50	51	101
D	NC 3	30	27	57
E	NC 4	30	69	99

# = Number, Ref = reference, PC = positive control, NC = negative control samples.

## Data Availability

Not available.

## References

[1] World Health Organization (WHO) Global Tuberculosis Program. https://www.who.int/teams/global-tuberculosis-programme/data.

[2] Flynn J.L., Chan J. (2001). Immunology of tuberculosis. Annu. Rev. Immunol..

[3] Timmins G.S., Deretic V. (2006). Mechanisms of action of isoniazid. Mol. Microbiol..

[4] Kim E.J., Kim J.H., Kim M.S., Jeong S.H., Choi D.H. (2021). Process analytical technology tools for monitoring pharmaceutical unit operations. Pharmaceutics.

[5] Igne B., Ciurczak E., Ozaki Y., Huck C., Tsuchikawa S., Engelsen S.B. (2021). Near-infrared spectroscopy in the pharmaceutical industry. Near-Infrared Spectroscopy: Theory, Spectral Analysis, Instrumentation, and Application.

[6] Crocombe R. (2018). Portable spectroscopy. Appl. Spectrosc..

[7] Bec K.B., Grabska J., Huck C.W. (2021). Miniaturized near-infrared spectroscopy principles and applications. Chem. Eur. J..

[8] Yan H., Siesler H.W. (2018). Hand-held near-infrared spectrometers: State-of-the-art instrumentation and practical applications. NIR News.

[9] Riba J.R., Puig R., Cantero R. (2023). Portable instruments based on BIR sensors and multivariate statistical methods for a semiautomatic quality control of textiles. Machines.

[10] Krauenburg R.F., Ramaker H.J., Sap S., van Asten A.C. (2022). A calibration friendly approach to identifying drugs of abuse mixtures with a portable near-infrared analyzer. Drug Test. Anal..

[11] United States Agency for International Development USAID Global Health Supply Chain. https://www.ghsupplychain.org/.

[12] Workman J.J. (2018). A review of calibration transfer practices and instrument differences in spectroscopy. Appl. Spectrosc..

[13] Eady M., Payne M., Sortijas S., Bethea E., Jenkins D. (2021). A low-cost and portable near-infrared spectrometer using open-source multivariate data analysis software for rapid discriminatory quality assessment of medroxyprogesterone acetate injectables. Spectrochim. Acta A.

[14] Varmuza K., Filzmoser P. (2009). Introduction to Multivariate Statistical Analysis in Chemometrics.

[15] Eady M., Payne M., Changpim C., Jinnah M., Sortijas S., Jenkins D. (2022). Establishment of instrumental operation qualification and routine performance qualification procedures for handheld near-infrared spectrometers used at different locations within a laboratory network. Spectrochim. Acta A.

[16] Kaale E., Hope S.M., Jenkins D., Layloff T. (2016). Implementation of 350–2500 nm diffuse reflectance spectroscopy and high-performance thin-layer chromatography to rapidly assess manufacturing consistency and quality of cotrimoxazole tablets in Tanzania. Trop. Med. Int. Health.

[17] Gemperline P.J., Boyer N.R. (1995). Classification of near-infrared spectra using wavelength distances: Comparison to the Mahalanobis distance and residual variance methods. Anal. Chem..

[18] Sortijas S. (2023). Github. PCA-MDR. http://github.com/sortijas/MDA-PCA.

